# Do the Effects of Secondary Prevention of Cardiovascular Events in PAD Patients Differ from Other Atherosclerotic Disease?

**DOI:** 10.3390/ijms160714477

**Published:** 2015-06-25

**Authors:** Pavel Poredos, Mateja Kaja Jezovnik

**Affiliations:** Department of Vascular Disease, University Medical Centre Ljubljana, Zaloska 7, 1000 Ljubljana, Slovenia; E-Mail: matejakaja@gmail.com

**Keywords:** atherosclerosis, preventive measures, cardiovascular events

## Abstract

Atherosclerosis is considered a generalized disease. Similar or identical etiopathogenetic mechanisms and risk factors are involved in various atherosclerotic diseases, and the positive effects of preventive measures on atherogenesis in different parts of the arterial system were shown. However, until know, great emphasis has been placed on the aggressive pharmacological management of coronary artery disease (CHD), while less attention has been devoted to the management of peripheral arterial disease (PAD), despite its significant morbidity and mortality. Data on the efficacy of preventive measures in PAD patients have mostly been gained from subgroup analyses from studies devoted primarily to the management of coronary patients. These data have shown that treatment of risk factors for atherosclerosis with drugs can reduce cardiovascular events also in patients with PAD. The effects of some preventive procedures in PAD patients differ from coronary patients. Aspirin as a basic antiplatelet drug has been shown to be less effective in PAD patients than in coronary patients. The latest Antithrombotic Trialists’ Collaboration (ATC) meta-analysis demonstrates no benefit of aspirin in reducing cardiovascular events in PAD. Statins reduce cardiovascular events in all three of the most frequently presented cardiovascular diseases, including PAD to a comparable extent. Recent studies indicate that in PAD patients, in addition to a reduction in cardiovascular events, statins may have some hemodynamic effects. They prolong walking distance and improve quality of life. Similarly, angiotensin enzyme inhibitors are also effective in the prevention of cardiovascular events in coronary, cerebrovascular, as well as PAD patients and show positive effects on the walking capacity of patients with intermittent claudication. In PAD patients, the treatment of hypertension and diabetes also effectively prevents cardiovascular morbidity and mortality. As PAD patients are at a highest risk of cardiovascular complications, the risk factors of atherosclerosis should be treated intensively in this group of patients. Most of the preventive measures, including the drugs used for prevention of CHD, are also effective in PAD patients.

## 1. Introduction

Atherosclerosis encompasses a broad spectrum of disease conditions of the circulatory system that include coronary heart disease (CHD), cerebrovascular disease (CVD) and peripheral arterial disease (PAD). Epidemiological studies have identified several risk factors for atherosclerotic disease, including hypertension, hyperlipidemia, smoking, diabetes, obesity and physical inactivity [1]. Therefore, the treatment of risk factors and life style modification play a key role in the prevention of atherosclerotic disease and its complications. Preventive measures, including pharmacotherapy, are important not only due to the disease’s morbidity and mortality rates, but also because of their impacts on the quality of life. Over the past decade, the data from trials and subgroup analyses indicate that treatment of patients with antiplatelet drugs, statins and angiotensin converting enzyme (ACE) inhibitors prevents the progression of local disease, reduces accompanied cardiovascular events and improves the prognosis of coronary, cerebrovascular and peripheral arterial occlusive disease [2]. However, it remains to be seen whether all of these measures have similar effects in different vascular territories.

While great emphasis has been placed on the aggressive pharmacological management of coronary artery disease, less attention has been devoted to the pharmacological management of CVD and much less to PAD, despite their significant morbidity and mortality. Although the prognosis of PAD is relatively benign regarding viability and survival of the affected limb, except in diabetics and patients with end-stage renal disease, all patients with PAD are at increased risk of myocardial infarction, ischemic stroke and cardiovascular death. Several studies describe a two- to three-fold greater mortality in patients with PAD than in age-matched controls with normal ankle-brachial index (ABI), with a five-year mortality of about 30% in patients with PAD. According to similar or identical etiopathogenetic mechanisms, the positive effects of the treatment of risk factors on atherosclerotic lesions in different parts of the vascular system are expected. However, some data show that the effect of drugs used in the prevention of cardiovascular disease is to some extent dependent on the various locations of atherosclerotic disease, and in patients with PAD, some preventive measures are less effective than in CHD patients [3].

All articles included in this review were identified by searching the MEDLINE and PubMed databases. We looked for the terms “cardiovascular events”, “acute coronary syndrome”, “peripheral arterial disease” and “ischemic stroke”, in combination with “atherosclerosis, prevention, pathophysiology, treatment”.

## 2. Antiplatelet Drugs

Antiplatelet drugs represent one of the basic options for the management of patients with various atherosclerotic diseases. Aspirin is the oldest and most often prescribed antiplatelet drug. The efficacy of aspirin depends probably on the type or location of atherosclerotic disease. It seems that it is most effective in coronary patients with clinically-unstable disease, and its efficacy is uncertain in PAD patients. One of the first meta-analyses (Antithrombotic Trialists’ Collaboration (ATC)) [3] indicated that antiplatelet drugs also significantly reduce cardiovascular events in patients with PAD. However, only one third of the PAD patients included in this meta-analysis were treated with aspirin, while the rest received other antiplatelet drugs.

A recent collaborative meta-analysis ATC [4] analyzed the effects of aspirin in primary and secondary prevention of vascular disease. Six primary prevention trials (95,000 individuals at low average risk) and 16 secondary prevention trials (17,000 individuals at high risk) were included. In this analysis, patients with PAD were not separately studied. In the primary prevention trials, aspirin only significantly reduced non-fatal myocardial infarction. The net effect on stroke was not significant, and vascular mortality did not differ significantly. In the secondary prevention trials, aspirin showed a great absolute reduction in serious vascular events with a non-significant increase of hemorrhagic stroke.

Meta-analysis of randomized trials of the efficacy of aspirin for the prevention of cardiovascular events in patients with PAD only investigated the effect of aspirin on cardiovascular event rates in patients with PAD only [5]. Randomized trials of aspirin therapy with or without dipyridamole involved 5269 individuals. The results of this meta-analysis demonstrated that for patients with PAD, aspirin therapy alone or in combination with dipyridamole did not significantly decrease the primary endpoints of cardiovascular events (non-fatal myocardial infarction, non-fatal stroke and cardiovascular death). Only a significant reduction in non-fatal stroke was observed.

The effect of aspirin on cardiovascular events in patients with preclinical PAD was studied in the Aspirin for Asymptomatic Atherosclerosis (AAA) trial [6]. This double-blind randomized controlled trial included 3350 subjects with a low ankle-brachial index (ABI) (≤0.95). Participants were followed for 8.2 years. No statistically-significant difference in endpoints was found between the subjects treated with aspirin (100 mg) and the placebo group.

## 3. Reasons for the Non-Efficacy of Aspirin in PAD Patients

Antiplatelet therapy reduces, but does not eliminate, the risk of ischemic events. It seems that non-responsiveness to aspirin is particularly expressed in patients with PAD. The reasons for non-responsiveness to aspirin in patients with PAD are various and include patients’ non-compliance, more advanced atherosclerotic diseases and pharmacogenetic reasons.

In contrast to the abundance of randomized trials evaluating aspirin and other antiplatelet drugs in the primary and secondary prevention of coronary heart disease, patients with PAD have been under-represented in randomized trails. Therefore, there is a lack of evidence-based data. Furthermore, the subgroup analysis of the PAD patients included in large controlled studies has failed to answer the question of the efficacy of aspirin in PAD patients, who were treated with very different dosages of aspirin and in combination with other antiplatelet drugs.

The efficacy of antiplatelet drugs is different in distinct patient populations and is dependent on the type and location of atherosclerotic disease [7]. In the Antiplatelet Trialists’ Collaboration [3], the greatest benefit was found in coronary patients, particularly in myocardial infarction and unstable angina pectoris patients. The antiplatelet treatment also significantly reduced the occurrence of recurrent ischemic stroke, albeit less than the occurrence of coronary events. Patients with peripheral arterial disease showed a 23% reduction of serious vascular events. These results show that aspirin is effective in the prevention of atherosclerotic cardiovascular events in all three most frequently affected vascular beds, but the greatest efficacy of aspirin was indicated in the coronary bed.

In the last decade, the concept of aspirin resistance as one of the most important reasons for aspirin non-responsiveness has been emphasized, despite the lack of any link between laboratory tests and clinical outcomes in patients. The genetic variability of COX-1 is responsible for the persistent increased production of platelet TXA2 in patients chronically treated with aspirin [8]. Aspirin resistance has been reported to range between 0% to as much as 59.5% [9]. However, it is not clear if there is a significant relationship between the *in vitro* and *in vivo* resistance to aspirin and how platelet activity as determined by different laboratory tests influences clinical outcomes. Recently, two meta-analyses were published, and their conclusions were that patients with aspirin resistance are at greater risk of recurrent cardiovascular events [9,10].

Advanced atherosclerosis, the greater atherosclerotic burden in this group of patients and, consequently, their more expressed inflammatory response and increased platelet activation are some of the potential reasons for the non-responsiveness of PAD patients to aspirin [11].

Mental or physical stress and systemic inflammatory response may also contribute to higher platelet reactivity [12]. Furthermore, the broad use of medications in cardiovascular patients, like statins, angiotensin receptor blockers and blockers of β adrenergic receptors, may inhibit aspirin activity and its clinical effect through different pathways.

## 4. Does an Alternative for Aspirin Exist in Patients with PAD?

Clopidogrel is an alternative antiplatelet agent that inhibits adenosine diphosphate (ADP)-induced platelet aggregation through the irreversible inhibition of P2-nucleotide receptors on the platelet surface. It is commonly used for secondary prevention of atherothrombotic disease in place of low-dose aspirin in patients who have experienced gastrointestinal intolerance, an aspirin-related adverse event. The efficacy of clopidogrel in comparison to aspirin was investigated in the Clopidogrel *vs.* Aspirin in Patients at Risk of Ischemic Events (CAPRIE) trial. In this trial, in the subgroup of patients with PAD, clopidogrel (75 mg daily) was found to be more effective than aspirin (325 mg daily) in preventing atherothrombotic events and resulted in fewer gastrointestinal bleedings. Among the 6452 patients with PAD, clopidogrel reduced the risk of myocardial infarction, stroke or vascular death by 23.8% more than aspirin [13]. Like aspirin, clopidogrel may cause adverse bleeding events. A meta-analysis of the adverse events of low-dose aspirin and clopidogrel showed that compared to clopidogrel, aspirin increases the risk of gastrointestinal bleeding, but not other bleedings. However, 883 subjects would need to be treated with clopidogrel instead of aspirin in order to prevent one major gastrointestinal bleeding episode annually [14].

Similarly, as was shown for aspirin, one of the most significant weaknesses of clopidogrel is its resistance, which seems to be clinically even more important than for aspirin. This non-responsiveness mainly depends on acquired or genetic changes in the activity of cytochrome P 450 (CYP-450) in the liver, which regulates clopidogrel metabolism [15].

The new antiplatelet drugs prasugrel, ticagrelor and picotamide seem to be more effective than aspirin in PAD patients, particularly in diabetic patients with PAD. A novel antagonist of protease-activated receptor (PAR)-1, to the primary receptor for thrombin on human platelets, on vascular endothelium and smooth muscle cells was used in patients with different atherosclerotic disease. In the study of Bonaca and co-workers, vorapaxar in PAD patients did not reduce the risk of cardiovascular death, myocardial infarction or stroke. However, vorapaxar significantly reduced the rates of hospitalization for acute limb ischemia and peripheral artery revascularization. The beneficial effects of PAR-1 antagonists on vascular events were accompanied by an increased risk of bleeding [16].

Therefore, antiplatelet drugs are effective in the prevention of cardiovascular events in various atherosclerotic diseases, including PAD. However, recent studies indicate that aspirin is less effective in PAD patients than in patients with coronary artery disease. New antiplatelet drugs showed marginal superiority over aspirin without definite advantages [15,17]. Aspirin thus remains the first-choice antiplatelet drug for secondary prevention of cardiovascular events in PAD patients, and clopidogrel is an effective alternative. Further, new studies on PAD patients are necessary to better define the role of antiplatelet agents in these patients, and personalized antiplatelet therapy is a promising way of accessing antiplatelet treatment.

## 5. Lipid Lowering

Various studies showed that hyperlipoproteinemia is also a relevant risk factor for PAD. Hypercholesterolemia was found in 45% to 59% of symptomatic PAD patients, and a significant benefit of statin treatment on cardiovascular and cerebrovascular events has been shown in this group of patients [18]. Routine statin use in vascular patients, either managed conservatively or undergoing open surgical or endovascular procedures, is associated with several beneficial effects [19]. Statins also exert several beneficial effects for vascular patients with abdominal aortic aneurysms, carotid artery disease, as well as arterial disease of the lower limbs [20]. A large meta-analysis, the Cholesterol Treatment Trialists’ Collaboratoration, showed that each 1.0-mmol/L decrease in low-density lipoprotein cholesterol by statin treatment is associated with a 12% reduction in all-cause mortality [21]. One of the most relevant studies on the efficacy of statins, the Heart Protection Study (HPS), showed that PAD patients treated with simvastatin noted a significant reduction in major vascular events, such as myocardial infarction, stroke and cardiovascular death, and needed revascularization less frequently [18]. In the group of patients with PAD only, the 24.5% proportional reduction in major vascular events in the simvastatin group in comparison to placebo was similar to the overt results of the full trial, which showed a 22% reduction in the event rate. Five years of statin treatment prevented 70 major cardiovascular events in 1000 patients with PAD. This very large randomized trial supports the use of statin drugs, not only in patients with coronary artery disease, but also in patients with arterial disease of other arterial beds. In another relatively small retrospective study, the use of statins substantially reduced vascular events in PAD patients [22].

Studies specifically addressing the outcome of PAD and the effect of local ischemic symptoms are sparse. In a subgroup of patients treated with simvastatin in the Scandinavian Simvastatin Survival Study (4S study), the relative risk of new claudication or worsening of pre-existing claudication was 0.6, as compared with patients randomly assigned to placebo with a relative risk reduction of 38% [23]. The effect of lipolysis therapy on intermittent claudication was also investigated in the Lipid Research Clinics Coronary Primary Prevention Trial [24]. Cholestyramine achieved a 15% reduction in claudication risk compared to placebo.

Statin use is also associated with better primary patency after infrainguinal by-pass graft surgery or endovascular treatment. The beneficial effects of statins were independent of the total cholesterol and LDL levels [25]. A large international cohort of patients with established PAD indicates that patients who were taking statins had a significantly lower risk of adverse limb and systemic cardiovascular outcomes. Among patients with PAD in the REduction of Atherothrombosis for Continued Health (REACH) registry, statin use was associated with an 18% lower rate of adverse limb outcomes, including worsening symptoms, peripheral revascularization and ischemic amputations [26]. Similarly, also positive effects of statin use in patients with an advanced form of PAD (critical limb ischemia) were confirmed in the study of Westin and co-workers. Statin therapy was associated with lower one-year rates of major adverse cardiovascular events, mortality, major amputation or deaths. Statin use was also associated with an improved patency of arteries among patients undergoing infrapopliteal angioplasty [27].

Evidence also suggests that statins reduce perioperative, as well as long-term morbidity and mortality in patients undergoing non-cardiac vascular surgery [28]. One of the meta-analyses shows that preoperative statin treatment considerably reduces postoperative mortality in comparison with statin non-use. Preoperative use of fluvastatin in patients undergoing abdominal aortic aneurysm repair or lower limb arterial reconstruction or carotid endarterectomy was associated with a reduction of postoperative myocardial ischemia and death from cardiovascular causes [29].

In PAD patients, statins probably had some additional (hemodynamic) effects and, independently of cholesterol reduction, improved walking distance and the quality of life. In the Treatment of Peripheral Atherosclerotic Disease with Moderate or Intensive Lipid Lowering (TREADMILL) study, prolongation of the claudication distance was noted in patients with intermittent claudication treated with 80 mg atorvastatin for 12 months [30]. Another study found improvement of the pain-free walking distance in PAD patients during treatment with 40 mg of simvastatin as early as after three months.

Therefore, lipid lowering therapy prevents cardiovascular events in coronary, cerebrovascular, as well as in PAD patients with comparable effects. However, in PAD patients, additional pleotropic effects of statins, such as improvement of pain-free walking distance and quality of life, have also been confirmed.

## 6. Managing Hypertension

Hypertension is one of the most important risk factors for atherosclerotic disease, including PAD. In patients with preclinical and clinical stages of PAD, which is present in 20% of the older population, hypertension was found in 50% to 92% [31]*.* In the Peripheral Arterial Disease Awareness, Risk, and Treatment: New Resources for Survival (PARTNERS) study, hypertension was more common among patients with PAD or cardiovascular disease than among the control subjects without clinically-recognized atherosclerosis [32]. Follow-up data from the Framingham Study found a 2.5- to 4-fold increased risk of PAD in men and women with hypertension [33].

Nevertheless, not all studies have demonstrated a clear relation between hypertension and PAD. For example, the Reykjavik Study found a low incidence of PAD in Icelandic men aged 60 to 70 years old, which was not related to the presence of systolic or diastolic hypertension [34].

However, most of the findings support that the presence of hypertension additionally increases the risk of cardiovascular events in PAD patients, as was confirmed in the SHEP (Systolic Hypertension in the Elderly Programme) study, where a low ankle-brachial index (<0.9) in an older population in conjunction with hypertension predicted a two- to three-fold increased risk of cardiovascular mortality [35]*.* Since hypertensive patients with PAD are at an especially high risk of cardiovascular events, intensive management of hypertension is urgently necessary.

There is ample evidence that antihypertensive treatment reduces the risk of cardiovascular events in PAD patients. In the ABCD (Appropriate Blood Pressure Control in Diabetes) trial, it was shown that there is a striking, inverse relationship between ABI and cardiovascular events. However, the influence of pathological ABI on cardiovascular events was completely eliminated by intensive blood pressure lowering, but not in the group with a moderate blood pressure decrease [36]*.* Limited data exist as to whether treatment of hypertension prevents the development of intermittent claudication. Only the TOMHS (The Treatment of Mild Hypertension) study showed an improvement in intermittent claudication during antihypertensive treatment [37]. In ALLHAT (Antihypertensive and Lipid-Lowering Treatment to Prevent Heart Attack Trial), blood pressure lowering had no effect on the development of PAD [38]*.*

## 7. Choice of Antihypertensive Drugs in PAD

Any class of antihypertensive drugs can be used to treat hypertension in most patients with PAD, including beta blockers. Although a meta-analysis of 11 randomized control studies showed that beta (β) adrenergic blocker therapy did not worsen intermittent claudication [39], starting the treatment of hypertension in PAD patients with β blockers can provoke the onset of intermittent claudication and vasospasms. β Blockers should be carefully prescribed to PAD patients with critical limb ischemia and vasospastic disorders [40]. Dihydropyridine calcium channel blockers are appropriate for hypertensive patients with PAD and accompanying vasospastic disturbances [40]. Carvedilol, which combines the blockade of β-1, β-2 and α-1 adrenergic receptors and has adjunctive antioxidant, anti-inflammatory and antiarrhythmic properties, is the preferred agent in PAD patients [41]*.* Nebivolol is a promising novel cardio-selective beta blocker that also promotes vascular nitric oxide production and improves endothelial function*.* However, in patients with PAD, there is no conclusive, outcome-related data favoring the use of carvedilol or nebivolol over other beta blockers.

ACE inhibitors exert various beneficial actions on the cardiac and vascular structure and function, beyond their blood pressure-lowering effects. These drugs improve endothelial function, cardiac and vascular remodelling, slow the progression of atherosclerosis and reduce the risk of cardiovascular events and death [42]*.* The antiatherogenic effect of the ACE inhibitor ramipril was confirmed in the Heart Outcomes Prevention Evaluation (HOPE) study, where ramipril provided equal relative protection against myocardial infarction, stroke and cardiovascular death in patients with PAD as in other high-risk groups [43]*.* The absolute benefit of ramipril was greater in patients with a reduced ankle-brachial pressure index than in those with a normal ankle-brachial pressure index, *i.e.*, 50 *vs.* 24 cardiovascular events were prevented per 1000 treated patients in the 4.5 years of follow-up*.* In the European Trial on Reduction of Cardiac events with Perindopril in Stable Coronary Artery Disease (EUROPA) study that included more than 12,000 patients with stable coronary artery disease, among which 883 were patients with PAD, all subgroups treated with perindopril, including the one with PAD, noted a similar reduction of cardiovascular events of about 20% [44].

ACE inhibitors in PAD patients may have some hemodynamic effects. Recently, a randomized controlled trial on the effect of ramipril on walking times and the quality of life in patients with intermittent claudication was published [45]. This study was conducted among 212 patients with intermittent claudication who were randomized to receive 10 mg/d ramipril or matching placebo for 24 weeks. It was shown that ramipril significantly increased pain-free and maximum treadmill walking time compared to the placebo. This was associated with a significant increase in the quality of life. The increase in walking times represent (77% and 123% increases in pain-free and maximum walking times, respectively) a greater improvement than was reported for other conventional drug therapies, including pentoxifylline and cilostazol.

## 8. Diabetes Management

Impaired fasting glucose concentration and diabetes are common in patients with PAD. Only 40% have a normal fasting glucose concentration [46]. Aggressive diabetes treatment reduces the risk of microvascular complications [47]. PAD patients with non-medicated diabetes show a higher rate of mortality and peripheral artery interventions compared to patients with normal fasting glucose levels [48]. The Retrospective Analysis of Diabetes Control and Complications Trial of 1441 type 1 diabetes patients found that intensive insulin therapy compared with conventional treatment decreased the risk of combined major macrovascular events by more than 40% during the average follow-up of 6.5 years, although the results were not statistically significant (*p =* 0.08) [49]. After 17 years of follow-up of the same cohort, the difference reached statistical significance (*p =* 0.02). The United Kingdom Prospective Diabetes Study of 3867 patients with type 2 diabetes found that treatment with sulfonylureas or insulin *vs.* diet-based treatment significantly reduced the risk of myocardial infarction by 16%, but the effects on stroke, limb amputation or death were not significant [50]. Levels of A1c hemoglobin were found to be positively associated with the risk for PAD in diabetics [51]. Diabetic patients with poor glucose control were more than five-times more likely to develop intermittent claudication than patients with good glucose control. Patients with oral hypoglycemics prescribed have outcomes similar to those of patients that did not have diabetes, and also, the mortality of these patients was similar to that of patients without diabetes. This agrees with the existing data, which indicate that oral hypoglycemics, particularly metformin, reduce the incidence of major adverse events in patients with cardiovascular disease [52]. It was shown that also elevated lipoprotein (a) level in diabetic patients with PAD is an indicator of poor prognosis [53]. In addition to glycemic control, the main factors of preventing macrovascular complications in diabetic patients are strict management of hypertension and dyslipidemia, administration of antiplatelet therapy and smoking cessation.

## 9. Conclusions

In conclusion, secondary prevention of cardiovascular events in different atherosclerotic diseases is mostly based on medical management of risk factors of atherosclerosis. In spite of some differences in the development of atherosclerotic process and complications in different parts of the arterial system, the basic pathogenetic mechanisms are similar or identical. Therefore, it is expected that the same preventive measures can be used in the prevention of the progression of different atherosclerotic diseases. Available data are mostly gained form the population of patients with CHD. However, subgroup analysis of large multicenter studies, as well as some new, recent studies confirmed that most of these preventive measures, including medication, are effective also in patients with PAD, who are at the highest risk for cardiovascular complications. However, the effects of individual drugs to some extent differ by the type and location of atherosclerotic disease. In PAD patients, classical antiplatelet drugs, like aspirin, are less effective than in CHD. Further, it was shown that other drugs that are most frequently used in the secondary prevention of atherosclerosis, like statins and ACE inhibitors, have in patients with PAD hemodynamic effects, improving the claudication distance and quality of life. Some of these drugs also, independently of prevention of cardiovascular events, prevent the progression of local disease and decrease the amputation rate of diseased leg.
